# Sexual function and pregnancy outcomes after periacetabular osteotomy: a systematic review

**DOI:** 10.1093/jhps/hnae039

**Published:** 2024-12-05

**Authors:** Katie Hutchinson, Khurram Baig, Christian Smith, Ajay Malviya

**Affiliations:** Department of Orthogeriatrics, Croydon University Hospital, 530 London Road, Croydon CR7 Y7E, UK; Department of Trauma and Orthopaedics, Newcastle University, Framlington Place, Newcastle NE2 4HH, UK; Department of Trauma and Orthopaedics, Guy’s and St Thomas’ Hospital, Great Maze Pond, London SE1 9RT, UK; Department of Trauma and Orthopaedics, Northumbria Healthcare NHS Trust, 8 Silver Fox Way, Newcastle upon Tyne NE27 0QJ, UK

## Abstract

Periacetabular osteotomy (PAO) is a surgical treatment of developmental dysplasia of the hip (DDH). This systematic review investigates the impact PAO has on sexual function and pregnancy through changes in patient-reported outcomes. A literature search of Medline, PubMed, EMBASE, and the Cochrane Library from 1996 to November 2023 was performed. Search terms included: ‘periacetabular osteotomy’, ‘pelvic osteotomy’, ‘outcomes’, ‘sexual function’, ‘pregnancy’, and ‘childbirth’. Assessment of methodological quality was performed using the methodological index for nonrandomized studies (MINORS) criteria and Grading of Recommendations, Assessment, Development, and Evaluations (GRADE) system. The search returned 560 papers, with 5 meeting the inclusion criteria. Sexual function improved over both short- and longer-term follow-up periods. 48.2% of patients had improved sexual function following PAO. Female patients reported greater improvement than males, who had a higher baseline score. No complications were documented in pregnancy or delivery compared to patients who had not had a PAO. Increased rates of caesarean sections (CSs) were seen in all studies (37.4%) compared to the European average. All five papers were calculated to be of low quality according to the MINORS criteria and low certainty according to the GRADE level of evidence. Patients receiving a PAO due to symptomatic DDH had improved sexual dysfunction due to a reduction in hip pain, but almost half of these patients may not appreciate any benefit. There is an increased likelihood of CS in patients post-PAO, with no reliable evidence for its necessity. Limitations of this review included poor quality and availability of evidence, as all studies were retrospective cohort studies. Further larger powered studies are required to assess the full impact of PAO on both sexual function and pregnancy.

## Introduction

Developmental dysplasia of the hip (DDH) is a structural abnormality of the hip joint that can result in pain, gait abnormalities, and an increased risk of early-onset arthritis [1]. DDH predominantly presents in young women of childbearing age. Surgical options for treating symptomatic DDH include a periacetabular osteotomy (PAO), in which the acetabulum is reoriented to provide sufficient coverage of the femoral head, restore the natural biomechanics, reduce chondral damage, and improve function.

A previous systematic review by Clohisy *et al*. concluded that PAO improves hip function in most patients over short- and medium-term follow-up periods [2]. Modern minimally invasive approaches have reduced the complication rates [3]. Good long-term survival of the hip is also well documented [4–6]. While general conclusions regarding the efficacy of a PAO have been well supported in the literature, there is a paucity of data on more specific outcomes. As this procedure’s patient cohort is largely made up of women of childbearing age, to whom sexual function and pregnancy are particularly relevant, outcomes should consider these factors. The lack of clinical evidence limits the ability to assess the PAO’s overall impact more comprehensively, with possible negative connotations on the decision-making process for the patient and healthcare provider.

This systematic review aims to identify the impact a PAO has on the sexual function of patients and to assess its effect on decisions made during pregnancy. This review will specifically look at how the procedure impacts future pregnancies and evaluate if there is evidence of an increased risk of pregnancy-related complications.

## Materials and methods

This systematic review was undertaken in accordance with the Preferred Reporting Items for Systematic reviews and Meta-Analyses (PRISMA) guidelines [7]. It was registered with the International Prospective Registry of Systematic Reviews (reg. no. 447935703321). The review was registered with Prospero [8].

### Search strategy

**Figure 1.**
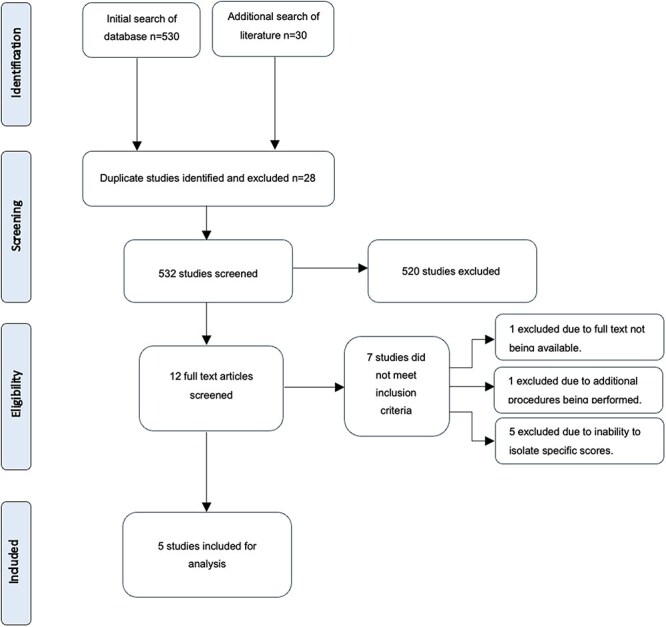
Flowchart of literature search.

A database search was performed on Medline, PubMed, EMBASE, and the Cochrane Library from 1996 to November 2023. Keywords searched included: ‘periacetabular osteotomy’, ‘pelvic osteotomy’, ‘outcomes’, ‘sexual function’, ‘pregnancy’, and ‘childbirth’. All abstracts identified by the search strategy were screened, and relevant papers were read in full. Two reviewers scrutinized full-text copies of the identified papers before inclusion, with any disagreements resolved by the senior author. Only papers focusing on postprocedural outcomes, including data on sexual function and pregnancy, were selected. This included studies that used scoring systems, including sections on sexual activity or pregnancy in assessing outcomes. This process has been detailed in the flowchart attached to this review (Fig. 1).

### Data extraction

Information about each of the studies was collected, including study type, level of evidence, sample size, sex distribution (M/F), average age at surgery, follow-up duration, indication, and treatment approach (Table 1).

**Table 1.** Demographics.

Paper	Author	Journal	Study size (M/F)	Average age at surgery (follow-up duration)	Indication	Intervention
Periacetabular osteotomy: sporting, social and sexual activity 9–12 years post surgery	Kilt *et al*. [11]	*HIP International*	52 (12/50)	31 (10 years)	Symptomatic acetabular dysplasia	Periacetabular osteotomy (ilioinguinal approach)
Is previous periacetabular osteotomy associated with pregnancy, delivery, and peripartum complications?	Bartosiak *et al*. [15]	*Clinical Orthopaedics and Related Research*	96 (0/96)	26.5 (3.9 years)	Symptomatic acetabular dysplasia (with or without femoroacetabular impingement); deformities from Legg–Calvé–Perthes disease	Periacetabular osteotomy
Birth after peri-acetabular osteotomy	Flückiger *et al*. [16]	*Orthopade*	93 (0/93)	N/A	N/A	Periacetabular osteotomy
Sexual activity, pregnancy, and childbirth after periacetabular osteotomy	Valenzuela *et al*. [12]	*Clinical Orthopaedics and Related Research*	122 (0/122)	32 (3.8 years)	Symptomatic acetabular dysplasia	Periacetabular osteotomy
Childbirth and sexual activity after eccentric rotational acetabular osteotomy	Masui *et al*. [13]	*Clinical Orthopaedics and Related Research*	21 (0/21)	25.7 (7.7 years)	Symptomatic acetabular dysplasia	Periacetabular osteotomy (eccentric)

Data regarding outcome measures were split into two groups: one focussing on sexual function outcomes and the other on pregnancy. Where papers focused on sexual function, data were collected pre- and post-PAO. In the pregnancy group, data were collected on the caesarean section (CS) rate and average birth weight, both of which served as valid endpoints for assessing the impact of PAO on pregnancy.

### Assessment of methodological quality

The quality of the papers included in this review was assessed using the methodological index for nonrandomized studies (MINORS) scoring system (Table 2) [9]. Only Questions 1–7 were relevant to the papers in this review, as Questions 8–12 were specific to comparative studies (three studies). Scores of 0–2 were ascribed for each of the seven relevant domains, producing a total score for each paper out of 14. A score of 0 is rated as poor, and 14 is rated as excellent.

**Table 2.** MINORS criteria.

Paper	Author	Journal	Level of evidence	MINORS score
Periacetabular osteotomy: sporting, social and sexual activity 9–12 years post surgery	Kilt *et al*. [11]	*HIP International*	IV	11/14
Is previous periacetabular osteotomy associated with pregnancy, delivery, and peripartum complications?	Bartosiak *et al*. [15]	*Clinical Orthopaedics and Related Research*	IV	11/14
Birth after peri-acetabular osteotomy	Flückiger *et al*. [16]	*Orthopade*	III	10/14
Sexual activity, pregnancy, and childbirth after periacetabular osteotomy	Valenzuela *et al*. [12]	*Clinical Orthopaedics and Related Research*	III	12/14
Childbirth and sexual activity after eccentric rotational acetabular osteotomy	Masui *et al*. [13]	*Clinical Orthopaedics and Related Research*	IV	10/14

**Figure 2.**
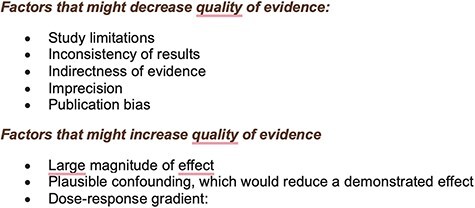
GRADE domains of quality.

**Figure 3.**
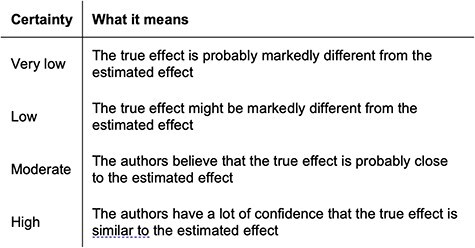
GRADE domains of certainty.

Further assessment of papers included was conducted using the Grading of Recommendations, Assessment, Development, and Evaluations (GRADE) framework [10]. Papers were classified depending on domains of quality (Fig. 2). It has four grades of recommendation: high, moderate, low, and very low (Fig. 3).

## Results

### Search results

The initial searches returned 560 articles (Fig. 1). Removal of duplicates left 532 studies. Twelve of the abstracts identified met the inclusion criteria. One paper was excluded due to the unavailability of full text. One paper was removed for recording additional surgical procedures. Five papers were excluded for failure to isolate specific sexual function scores from total data.

### Methodological quality

All five studies were retrospective cohort studies. Table 2 summarizes the results of the MINORS criteria [9]. The quality of the evidence was moderate, with an average score of 10.8 out of 14 (range 10–12). According to the GRADE scoring system, all five papers included in this review were of low certainty.

### Literature review—sexual function

All studies had a similar endpoint and shared a common theory regarding how PAO can influence sexual function via the prevention of progression to secondary arthritis. They discussed how increased hip stability following PAO surgery would increase the likelihood of successful pregnancies.

Klit *et al*. presented a retrospective cohort study that assessed quality of life and functional outcomes following PAO [11]. All patients in this study had symptomatic hip dysplasia. The authors created a questionnaire regarding outcomes, assessing sexual dysfunction and functional mobility. Only patients with preserved hip joints at follow-up were included. The mean follow-up was 9 years post-PAO. Fifty-two patients with a mean age of 31 years completed and returned the questionnaire, of which 72% were women. Data collected showed that sexual function improved in males and females post-PAO. In females, scores rose from 4/6 preoperatively to 5/6 postoperatively (*P* = 0.008). In males, improvement was from 5.5/6 to 6/6 (*P* = 0.102); this, however, did not reach statistical significance. Klit *et al*. was the only study to include male patients.

Valenzuela *et al*. is a retrospective cohort study focussed specifically on the impact of PAO on sexual function, pregnancy, and childbirth [12]. All patients had confirmed hip dysplasia. The aims of this study were congruent with this review and provided the most specific data regarding sexual function and pregnancy post-PAO. Ninety-four patients with a mean age of 32 years returned adequately completed questionaries and met the inclusion criteria [two excluded due to conversion to total hip arthroplasty (THA)], with an average follow-up of 3 years post-PAO. Seventy (74.5%) patients reported that they experienced no change in satisfaction with sexual intercourse, while 24 (25.5%) did report a change. Of the 24 patients that reported a change, 13 patients attributed it to reduced pain (54.2%), and 41.6% (10/24) reported improved range of motion.

Older patients (mean age 38 years old) had less pain than younger patients (mean age 30 years old) postoperatively. Older patients had a greater change in the frequency of sexual activity. Patients who changed sexual positions more frequently were significantly younger (30 years old compared to 37 years old, respectively).

The study by Masui *et al*. is a retrospective cohort study that focussed on the impact of PAO on childbirth and sexual activity [13]. PAOs were performed in patients with pain in the hip secondary to hip dysplasia or Legg–Calvé–Perthes disease. Patients were followed up for a minimum of 2 years postsurgery. This study had many similarities to the paper by Valenzuela *et al*., with a common aim and questionnaire [12]. Nineteen patients with a mean age of 30 years at the time of first childbirth returned adequately completed questionnaires and met the inclusion criteria with an average follow-up of 7 years post-PAO [13]. Two (10.5%) patients reported that they experienced no change in satisfaction with sexual intercourse, while 17 (89.5%) did report a change. This study stipulated whether the changes in satisfaction reported were positive or negative. Satisfaction with sexual activity improved in 68.4% (13/19) of patients but decreased in 21.1% (7/19). Patients were asked to record the reason for the changes and satisfaction with sexual intercourse. The most commonly reported reason was reduced pain 7/19 (41.2%). Within the group that reported an improvement in sexual function, the most reported reason was a reduction in the amount of hip pain they were experiencing. Four patients (21.1%) reported worsening sexual activity, which was attributed to reduced range of motion post-PAO.

Klit *et al*. reported a net improvement in sexual function in both males and females [11]. However, only the improvement seen in females was statistically significant. The authors discussed the reasoning behind the lack of statistical significance in males and identified a higher average male baseline as a significant contributory factor. This could also be a function of study design with a six-point scoring system limiting the ability of males to express the full magnitude of any gain in function. The other two papers consisted of female-only cohorts; hence, there was no comparison between the studies on male impact. The study by Klit *et al*., Valenzuela *et al*., and Masui *et al*. all excluded patients who went on to have THA. The exclusion of these patients is important, as receiving a THA soon after PAO implies little symptomatic improvement or functional gain.

Valenzuela *et al*. presented an in-depth assessment of how PAO impacts sexual function, yet despite all the data they collected, there was no clear, definitive answer to whether the impact was positive or negative [12]. Of their cohort, 89% was satisfied with surgery. However, only 25% reported a change in their satisfaction with sexual activity. The study design allowed patients to report multiple reasons as to why sexual satisfaction changed, which made the comparison of data challenging.

The study by Masui *et al*. reported the smallest sample size (21 patients) and only included patients who had delivered children post-PAO [13]. All the patients received a modified PAO called an eccentric rotational acetabular osteotomy. This is similar to the Bernese PAO and is an alternative method of improving femoral head coverage [14]. There is limited literature comparing the two approaches; however, studies have reported similar outcomes. Results associated with this study are primarily influenced by the surgical approach used on these patients. When comparing the results between studies, some heterogeneity could be inferred due to the differing osteotomy approaches.

A major contributor to heterogeneity between studies can be attributed to study design. Demographics were similar across all studies, primarily including young women of childbearing age. Differences in results stemmed primarily from the approach to data collection due to different questionnaires being used to assess outcomes in each study. Evaluation of outcomes was a notable limitation, resulting in a lack of standardization of an endpoint. Klit *et al*. collected scores on patient-reported sexual function before and after surgery, showing an improvement post-PAO. The paper by Klit *et al*. also did not provide raw data, which did not allow for further subanalysis. Valenzuela *et al*. and Masui *et al*. provided results that were harder to interpret and unclear in illustrating how sexual function had changed. More importantly, the paper by Valenzuela *et al*. failed to make clear whether there was a net improvement in sexual function or not. It was assumed that a reduction in pain and improved range of motion inferred improved sexual function. Due to this not being explicitly stated, there may be some confounders in the data.

According to the GRADE scoring system, the level of evidence and strength of recommendation was low. Study design limitations and imprecision largely contributed to the lower scores. All studies were retrospective cohort studies and, therefore, were not blinded. Outcomes were subjective and susceptible to bias. Small sample sizes contributed to a greater degree of uncertainty in results.

The three papers pertaining to sexual function were all low evidence and strength according to the GRADE system. One paper did not explicitly state whether there was a net improvement in sexual function, leaving it open to interpretation. One paper did report a significant improvement in sexual function; however, it had the smallest sample size. In total, 193 patients answered questionnaires pertaining to sexual function, with 93 patients (48.2%) indicating improved sexual function post-PAO. This was primarily attributed to the reduction of pain in 46.5% (20/43) based on the results of Valenzuela *et al*. and Masui *et al*.

Overall, the collective body of evidence suggests that PAO has a beneficial impact on sexual function, as supported by the findings of Klit *et al*. and Masui *et al*. Inferences from these findings must concede that the GRADE system scored the evidence as weak. The results are most relevant to women of childbearing age, as this constitutes most of the cohort included in this review. Males may not appreciate similar benefit as they do not seem to be as affected as females. The papers by Klit *et al*. and Masui *et al*. were given additional weight for their direct insight into the research question.

### Literature review—pregnancy

A total of four papers met the inclusion criteria. All studies reported the number of CSs observed within their respective cohorts, which served as a valid endpoint in assessing the impact of PAO on pregnancy. This was used as a standard measure, facilitating comparison between studies.

Bartosiak *et al*. included 31 patients with a mean age of 26.5 years at the time of surgery [15]. There were 38 successful pregnancies following PAO. The study found no difference in the rate of complications, preterm delivery and birth weight between this cohort and the population average. Delivery via CS was more likely in this cohort, with a rate of 53% (20/38 live births) and an average birth weight of 3218 g. The inclusion criteria of the study by Bartosiak *et al*. resulted in a cohort of patients who all had simultaneous procedures alongside PAO. The impact of these procedures on future pregnancies is an unknown variable that will have naturally influenced the data collected.

Flückiger *et al*. presented a retrospective cohort study, which included 17 female patients who had 28 successful pregnancies [16]. The study reported a CS rate of 36% (10/28) and an average birth weight of 3393 g. The reported rate is double the national average for CSs [17]. Both of these studies compared cohort averages against population data. Flückiger *et al*. were based in Germany, whereas the other studies used data from the USA. This is an important consideration when comparing qualitative outcomes.

Valenzuela *et al*. reported that 16 women went on to have 23 future pregnancies but only included data on 20 births, leading to the assumption that only 20 of these were full-term pregnancies. There was a CS rate of 23% (3/13) among patients with one pregnancy and 28% (2/7) among those with two, with an average of 25% (5/20) overall. The average birth weight was 3520 g [12]. Masui *et al*. reported that 21 women went on to have 29 full-term pregnancies. The reported CS rate was the lowest of all included studies at 27.5% (eight births). This study did not report data on the average birth weight.

It was commonly reported that there were concerns from healthcare staff regarding a patient’s suitability for a vaginal delivery after receiving a PAO. Flückiger *et al*. reported that in 50% of cases, the physician recommended the patient have a CS, with the sole justification being the history of PAO [16]. Other included studies did not explicitly state any medical or surgical reason as to why a CS was chosen. This raises questions regarding physician education on the management of patients post-PAO and the possibility that CSs may be overprescribed. Flückiger *et al*. investigated whether PAO led to any anatomical changes in the birth canal that would give rise to a surgical indication for CS. Their study concluded that there was no change in the birth canals of the women who went on to give birth, suggesting that CS may not have been indicated in this group of patients.

The GRADE system scored all four papers specific to pregnancy as of both low evidence and strength. It was apparent that there was a generalized trend towards higher rates of CS. In total, 37.4% (43/115) of all patients in this review delivered via CS. According to the World Health Organization, the average CS rate in Western Europe is 24.2% (95% confidence interval 18.3–30.2) [18]. All studies included in this review had higher elective CS rates than the national average [19]. Overall, the body of evidence produced consistent results with respect to the impact of PAO on pregnancy, and it can be concluded that despite the GRADE scoring, there is good evidence to suggest that in the PAO cohort, there is an increase in the CS rate with no clear indication for doing so.

## Discussion

The results of this review show an improvement in sexual function following PAO and no significant increase in pregnancy-related complications; however, there is an increased likelihood of delivery via CS. There is limited literature directly assessing sexual function following PAO; however, cohort studies such as Clohisy *et al*. reported showed that there is a significant improvement in pain, function, and quality of life [20]. It would be reasonable to assume that improvements in pain and function would relate to improved sexual activity. Males had a smaller improvement in sexual function postsurgery; however, they also had a greater average sexual satisfaction preoperatively compared to females. A similar finding was found following hip arthroscopy [21].

The results on the impact of PAO on pregnancy were consistent across the studies included in this review, with no associated major complications, average birth weight, and standard preterm delivery rates being reported in all studies. The most notable finding is the link between PAO and increased CS rates. The average CS rate following PAO across the studies was 37.4%. All studies included bar one reported a higher CS than the population average. Despite the small number of studies included, there is a clear trend towards patients delivering by CS, which may not be indicated.

Further studies are required to establish the extent of this significance. Klit *et al*. and Flückiger *et al*. observed that counselling from healthcare staff involved with the pregnancy was a common reason behind the decision for CS, with the primary indication being a history of PAO. There is no evidence to suggest that a PAO results in anatomical changes to the birth canal and it should not be a contraindication for vaginal delivery [11, 16]. Full maternal and foetal assessment should be performed on a case-by-case basis to establish the optimum method of delivery.

One of the lowest-scoring papers according to the MINORS criteria was the study by Flückiger *et al*., which was only available in German [16]. This is an important consideration, as despite being converted into English, it limited the interpretation of the paper. This did not have a bearing on quantitative data, which was still clear and accurately interpreted for the purpose of this review. This study included measurements of a patient’s birth canal pre- and post-PAO. The study remarked how there was no change in anatomical diameters of the birth canal, and therefore a PAO is not an indication for a CS. The paper was also written by Professor Reinhold Ganz, who performed the first PAO in 1984.

Overall, there is a low quality and quantity of literature assessing the influence of PAO on childbirth and sexual function. A similar systematic review evaluated the impact of different osteotomies on birth canal anatomy [22]. This review concluded that there were no changes to pelvic diameter with PAO, which would account for the increased CS rate. The increased CS rate was attributed to physician preference rather than anatomical requirements.

### Limitations

Several limitations were identified in this review. A major limitation was the limited literature and quality of evidence. All included papers were retrospective cohort reviews, which opened them to a large degree of bias due to researchers relying on individuals to recall specific variables surrounding their care potentially years prior [11, 15]. This could be addressed by the introduction of prospective studies and the collection of data from the day of diagnosis to postoperatively, allowing outcomes such as improvement in sexual function and pain to be assessed more thoroughly. Larger scale studies, such as randomized controlled trials, would provide improved quality data, and future reviews should look to include these data should it become available.

A significant limitation affecting all studies, and the literature in general, was the lack of a common approach to assessing outcomes relevant to the young female cohort. This was addressed through researchers coming up with their own questionnaires to collect relevant information on sexual function and pregnancy-related outcomes. This heterogeneity in how data were collected made the results of the studies hard to generalize or transform into a standard outcome measure. The creation of a standardized questionnaire for further assessment of outcomes would facilitate better data collection and analysis.

The small size of the cohorts included in each study was a well-documented limitation. In order for a full assessment of the impact of PAO on sexual function and pregnancy, further research with more significant sample sizes is required. Further research should be conducted to add statistical weight to the improvement of sexual function post-PAO.

## Conclusion

Sexual dysfunction caused by symptomatic DDH is improved after a PAO due to a reduction of hip pain, particularly in females. Although there is a trend towards improved sexual function (48.2% reported improvement), the poor-quality data may mask the true magnitude of the actual effect. Almost half of the predominantly female population undergoing PAO may not appreciate benefit in sexual function.

The rate of CS seems to be higher in the PAO group as compared with the general population, but there is no evidence to suggest that patients post-PAO are contraindicated from vaginal delivery. Further education should be distributed among healthcare providers to ensure patients are fully informed of their choices for childbirth. Large-scale standardized studies are required to fully establish the causality between improvements in sexual function and PAO.

## Data Availability

The data underlying this article are available in the article and in its online supplementary material.
